# Supplementary material for: Sodium Hexametaphosphate Serves as an Inducer of Calcium Signaling
Source: Biomolecules. 2023 Mar 23;13(4):577. doi: 10.3390/biom13040577 (PMC10135451; doi:10.3390/biom13040577)
Supplement: Supplementary file 1 [file biomolecules-13-00577-s001.zip › Supplemental Figure S1.pdf]

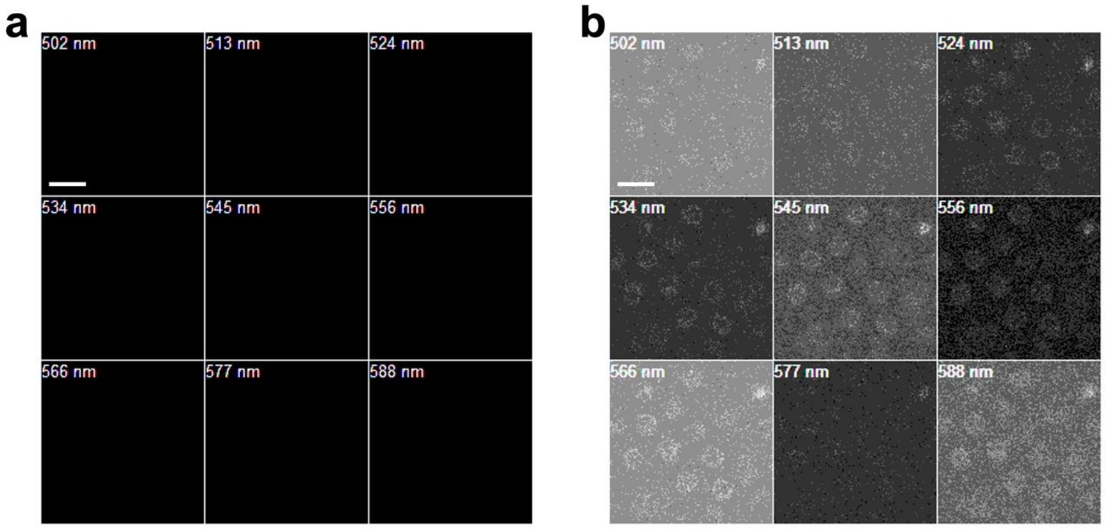

Supplemental Figure S1. **Detection of phosphate polymers by DAPI staining and electrophoresis. a,** Unstained oocytes served as negative controls. **b,** Images with enhanced contrast and brightness. Scale bars, 100 μm.
