# Supplementary material for: Sodium Hexametaphosphate Serves as an Inducer of Calcium Signaling
Source: Biomolecules. 2023 Mar 23;13(4):577. doi: 10.3390/biom13040577 (PMC10135451; doi:10.3390/biom13040577)
Supplement: Supplementary file 1 [file biomolecules-13-00577-s001.zip › Supplemental Figure S2.pdf]

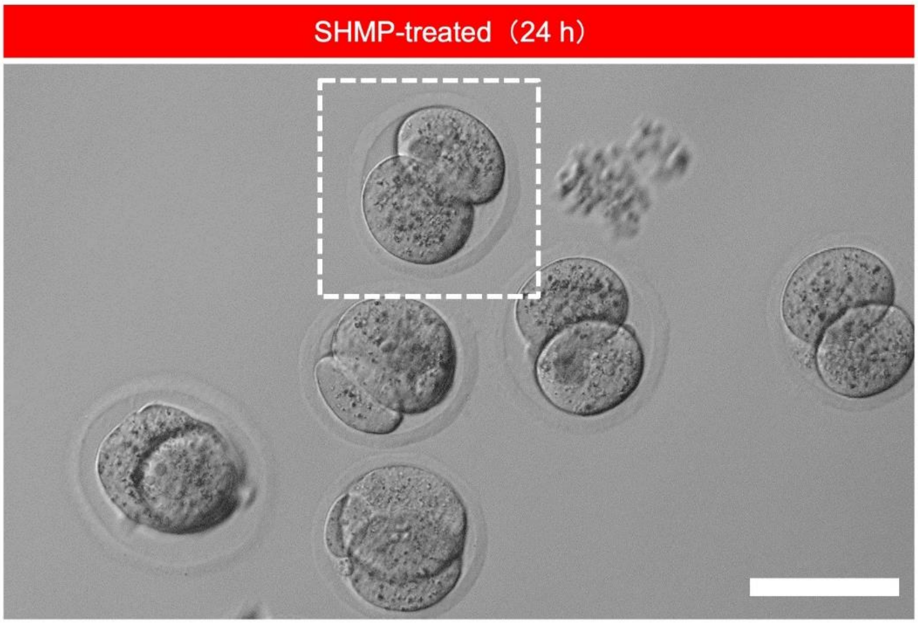

Supplemental Figure S2. Bright-field images of oocytes after SHMP treatment. The dotted box is enlarged in Figure 3b. Scale bar, 50 μm.
